# THE VALUE OF INTERNSHIPS IN CREATING AN AGE-SAVVY WORKFORCE

**DOI:** 10.1093/geroni/igad104.0487

**Published:** 2023-12-21

**Authors:** Laura Donorfio

**Affiliations:** University of Connecticut, Waterbury, Connecticut, United States

## Abstract

As the 65+ population continues to grow, so too does the need for an age-savvy workforce. There is not a field or profession that will not intersect in some way with older adults (Karasik, Donorfio, & Greenberg, 2023). Current work shortages and forecasts, however, raise considerable concern for a sustainable and adequately prepared workforce. With regard to preparation, gerontological field placements, internships, and practicums are becoming increasingly instrumental to workforce development. Both the Gerontology Competencies for Undergraduate and Graduate Education (2020) and AGHE’s Gerontology and Geriatric Curricular Standards and Guidelines (2021) strongly recommend internship, practicum, and/or field work experiences for students at all levels. Fostering a successful intergenerational experience, however, can be challenging. In this presentation, key components, core elements, necessary steps, best practices and logistical considerations will be discussed. For example, who are the key players and what are their roles in such experiences? What needs to happen at the programmatic and curricular levels? In addition to exploring the various facets of gerontological field placements, attention will be given to utilizing AGHE’s Standards and Guidelines and Gerontology Competencies to help identify several “interactional competencies” that relate to internships, practicums, and field experiences. This session also serves as an introduction to the range of perspectives (student, faculty, community agency) that follow.

